# A histopathological image classification method for cholangiocarcinoma based on spatial-channel feature fusion convolution neural network

**DOI:** 10.3389/fonc.2023.1237816

**Published:** 2023-08-18

**Authors:** Hui Zhou, Jingyan Li, Jue Huang, Zhaoxin Yue

**Affiliations:** ^1^ Department of Network Engineering, College of Computer and Software, Nanjing Vocational University of Industry Technology, Nanjing, China; ^2^ The Affiliated Changzhou No.2 People’s Hospital of Nanjing Medical University, Changzhou, China

**Keywords:** cholangiocarcinoma, histopathological image classification, convolution neural network, multiscale, feature fusion, feature reuse

## Abstract

Histopathological image analysis plays an important role in the diagnosis and treatment of cholangiocarcinoma. This time-consuming and complex process is currently performed manually by pathologists. To reduce the burden on pathologists, this paper proposes a histopathological image classification method for cholangiocarcinoma based on spatial-channel feature fusion convolutional neural networks. Specifically, the proposed model consists of a spatial branch and a channel branch. In the spatial branch, residual structural blocks are used to extract deep spatial features. In the channel branch, a multi-scale feature extraction module and some multi-level feature extraction modules are designed to extract channel features in order to increase the representational ability of the model. The experimental results of the Multidimensional Choledoch Database show that the proposed method performs better than other classical CNN classification methods.

## 1 Introduction

Cholangiocarcinoma (CCA) is an aggressive malignant tumor originating from the epithelium of the biliary system. Its incidence has been increasing over the last few decades (1, 2). Despite the advances in diagnosis and treatment, cholangiocarcinoma remains a devastating cancer with a 5-year overall survival rate of only 6.8%, and this value has not changed in recent decades (2). Available studies have shown that parasitic liver flukes, primary sclerosing cholangitis, chronic viral hepatitis B and C, bile duct cysts, hepatobiliary stones, and toxins are all risk factors for the development of cholangiocarcinoma (3). Current diagnostic methods for cholangiocarcinoma include ultrasound, CT scan, MRI, fluorodeoxyglucose positron emission tomography (FDG-PET), histopathological examination, and tumor marker testing. Although it has been suggested that the diagnosis of cholangiocarcinoma may be based on a combination of clinical presentation, laboratory analysis, and radiological assessment, most patients require a pathological diagnosis to confirm the diagnosis because radiological studies are nonspecific (4–6). In conclusion, the study of the histopathological examination of cholangiocarcinoma is essential and of great interest.

Traditional pathological diagnosis is a pathologist’s visual examination of a histological specimen under a microscope to determine whether a pathological section contains abnormal tissue. When the above process is digitally transformed (7), pathologists can remotely analyze pathological sections on a computer screen. Currently, histopathology is still largely a manual process (8). Pathologists’ diagnostic performance is based on their expertise and can be affected by decreased attention span due to fatigue. In order to reduce the workload of pathologists and improve the accuracy of diagnosis, it is of great clinical importance to develop an automatic classification algorithm for histopathological images. In the early stages of research, researchers commonly used two-stage image processing to develop algorithms. First, a set of features specific to that image type is extracted from the image using a series of one or more hand-crafted feature descriptors, which are then used to train the classifier (9). It should be noted that while this strategy is very commonly used in leukemia, breast cancer, and oral cancer (10–14), the accuracy of the classification task is highly dependent on the design and robustness of the specific feature extraction algorithms used. This means that expert knowledge and complex feature engineering are required to obtain reliable discriminatory features.

In recent years, it has been shown that deep learning (DL) overcomes these challenges (15). It can automatically abstract feature information in images from shallow to deep and achieve better classification results (16). In past biomedical competitions, Convolutional Neural Networks (CNN) and a variety of other DL-based algorithms have shown strong performance and achieved excellent results that surpass traditional algorithms (17, 18). Pre-training is a common tool for histopathological image classification. For example, Abunasser et al. (19) used the pre-trained ResNet50 model as a feature extractor and swapped a new densely connected classifier for prediction. In (20), a two-layer deep neural network has been proposed to classify breast cancer using the extracted features of a pretrained VGG16 model feature extractor from breast ultrasound images. Although these methods have made some progress, they have neglected the channel features in histopathological images. In addition, due to the wide variation in the morphology and size of biological tissues in histopathological images, it is difficult to extract discriminative features for classifying biological tissues with the previous methods. Based on this, this paper outlines a spatial channel feature fusion convolutional neural network (DCFCNN) for histopathological image classification of cholangiocarcinoma to assist pathologists in diagnosing malignancy. The proposed work contributes as follows.

1. This paper presents a classification approach for cholangiocarcinoma pathological images based on channel feature fusion that classifies cholangiocarcinoma pathological images using both spatial and channel data. Experimental results on a multidimensional cholangiocarcinoma histopathology dataset show that the fusion of spatial features and channel features can effectively improve the classification performance of the model.
2. In order to cope with the problem of large variations in cancer areas in histopathological images, this paper develops a Multiscale Feature Extraction (MSF) module to enhance the extraction of channel features. The results of the feature map visualization show that the method can significantly enrich feature information and reduce meaningless features, leading to better classification results.
3. To extract more representative channel discriminatory features, a Multi-Level Feature extraction module (MLF) was developed. Based on 1×1 convolution and feature reuse, the module can improve model representation by reusing channel features extracted from different levels of convolution to achieve better classification results.

The rest of the paper is organized as follows: The proposed methodology is detailed in Section 2. Experimental results and model evaluation are given in Section 3. Finally, conclusions and future work are presented in Section 4.

## 2 Proposed method

This section provides details of the proposed method. First, the Multidimensional Choledoch Database used to verify the method is described. Then the overall structure and detailed parts of the proposed method are described.

### 2.1 Dataset

**Figure 1:**
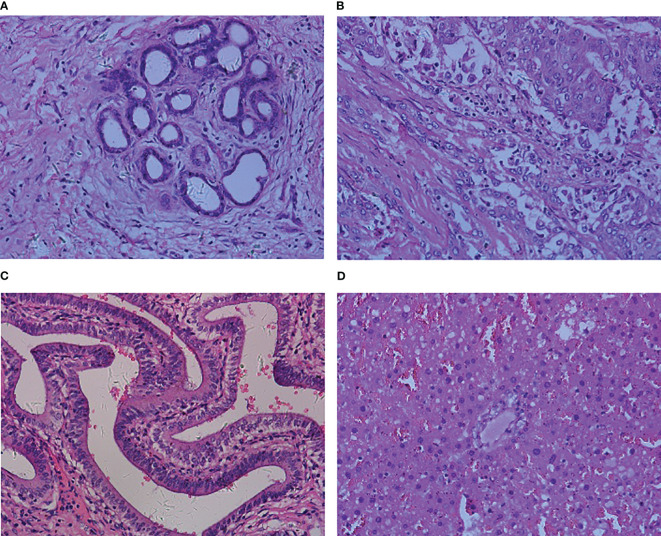
Multidimensional Choledoch Database (RGB images): **(A, B)** malignant samples, **(C, D)** benign samples.

The dataset used in this paper was derived from the Multidimensional Choledoch Database (21). It was created by the Shanghai Key Laboratory of Multidimensional Information Processing, East China Normal University in China. The choledoch tissues are collected at Changhai Hospital, Shanghai, China, with permission from the Ethics Committee. Each choledoch tissue is stained with hematoxylin-andeosin and the slide thickness is 10 µm. Among all slides, there are a total of 880 effective scenes of images. Note that all these images are collected using a magnifying factor of 20× and are acquired in two formats: 3-channel RGB, 8-bit depth in each channel, with a spatial dimension of 2304 ×1728 pixels, and 30-channel microscopic hyperspectral images, 16-bit depth per pixel, with 1280×1024 pixels per channel. For the qualitative comparisons, the RGB and microscopic hyperspectral images are taken in the same field of view. The images of the former format are used for experiments in this paper. In these multidimensional scenes, 690 scenes from 125 patients contain parts of cancer areas, 48 scenes from 14 patients are filled with cancer areas, and 142 scenes from 35 patients contain no cancer areas. We combine complete cancer region scenes and scenes partially containing cancer regions into one image class, which in turn transforms the problem into a binary classification problem of 738 malignant samples with images of cancer regions and 142 benign samples without cancer regions. **Figures 1A–D** shows four samples from the Multidimensional Choledoch Database.

### 2.2 Framework

**Figure 2:**
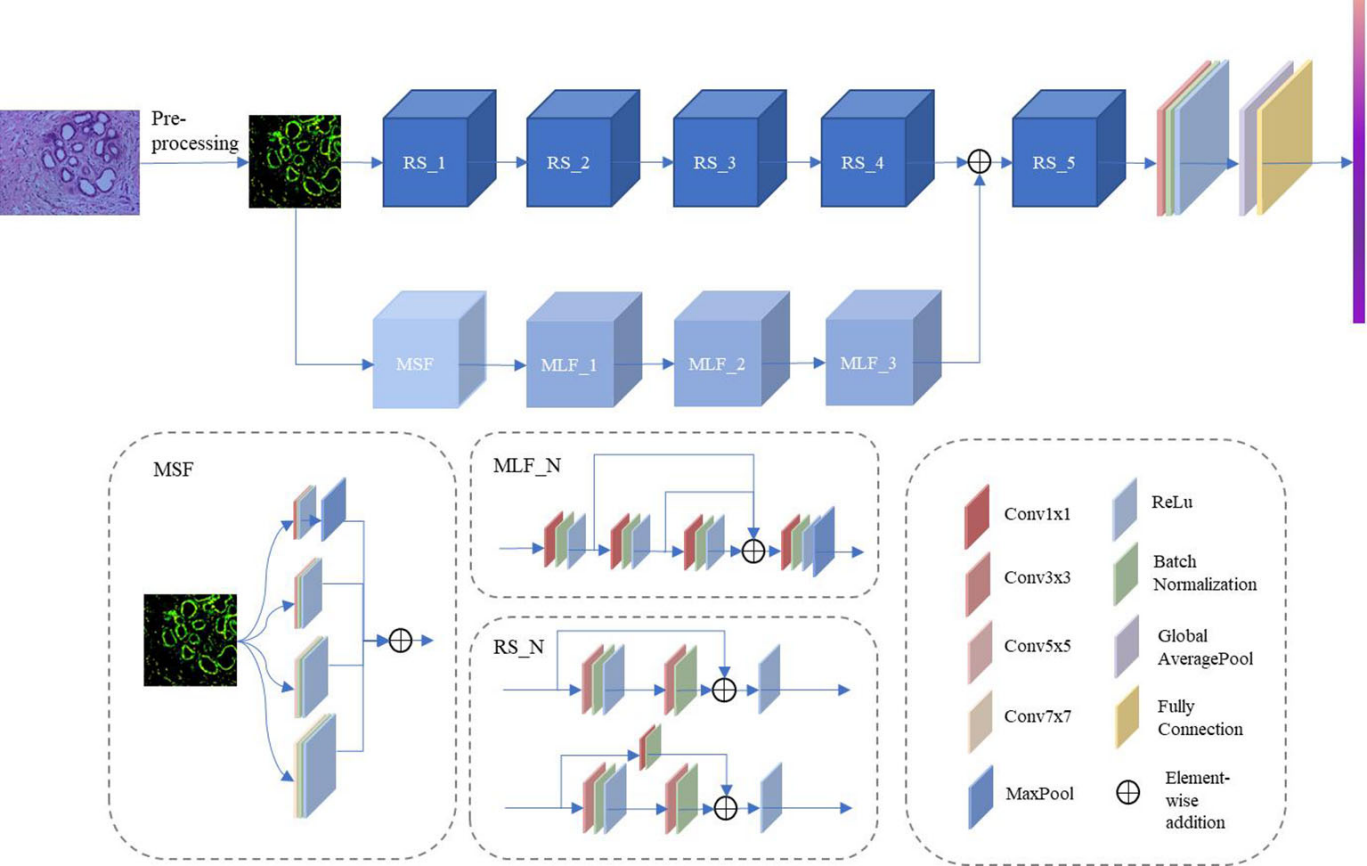
Framework of the proposed DCFCNN-based histopathological image classification of cholangiocarcinoma method.

Several previous studies have demonstrated the extraordinary performance of CNN in histopathological image classification tasks (21, 22), but most of these approaches are based on classic network architectures with insufficient classification performance. Inspired by the above approach, this paper outlines a novel DCFCNN for histopathological image classification of cholangiocarcinoma. **Figure 2** illustrates the framework of the proposed method. As shown in the figure, the input image is first pre-processed. Due to the large spatial dimensionality in histopathological images of cholangiocarcinoma, dimensionality reduction of the images is required to reduce the computational cost and to ensure that the algorithm is not interrupted by the memory limitations of the device (23). Therefore, the proposed method first reduces the spatial dimensionality of the input image by resizing and center cropping, and then normalizes and standardizes the reduced image to accelerate the convergence of the model. The processed images are then fed to DCFCNN to train the network. Finally, the trained network model predicts the pathological images of cholangiocarcinomas in the test dataset. The structure of DCFCNN includes the spatial branch and the channel branch. The spatial branch consists of 5 residual layers (RS_1-5), which are responsible for the extraction of the deep spatial features in the image. The channel branch consists of the MSF and 3 MLFs, which are responsible for extracting the channel features in the image. In addition, batch normalization is applied after the convolution layer and before the activation function to speed up the convergence of the network. The details of the RS, MSF and MLF structures are shown in **Figure 2**.

#### 2.2.1 RS

**Figure 3:**
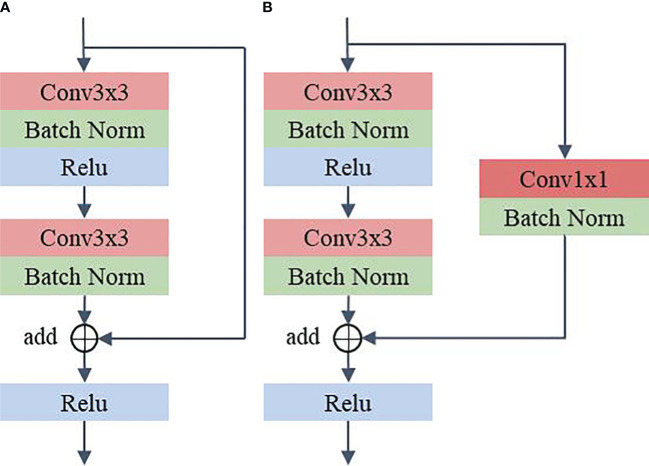
Residual structure. **(A)** Shortcut connection does not contain 1x1 convolution **(B)** Shortcut connection contain 1x1 convolution.

RS consists of residual structures (24) that are good at mitigating the degradation occurring in deeper networks and are able to extract discriminating features in deeper layers. Among these residual layers, RS_1 to RS_4 contain two residual structures. RS_5 contains three residual structures. **Figure 3** illustrates the specific parameters of the residual structure. As can be seen from the figure, each residual block contains two layers of convolution with 3x3 kernels and a shortcut connection (also known as a skip connection). Furthermore, in the residual structure shown in **Figure 3A**, the 3x3 kernel’s stride is 1 and padding is 1 to maintain the consistent dimensionality of the input and output feature maps. In the structure shown in **Figure 3B**, the 3×3 kernels in the first convolution layer have a stride of 2 and padding of 1. The 3×3 kernels in the second convolution layer have a stride of 1 and padding of 1. The feature map, after going through the structure shown in **Figure 3B**, halves the spatial dimension and doubles the channel dimension. In addition, in the shortcut connection shown in **Figure 3B**, a convolution layer with a 1×1 kernel is used to ensure dimensional matching. Compared to using the maxpool operation, **Figure 3B** can mitigate the loss of features due to downsampling. In the method proposed in this paper, the first residual structure in each residual layer is shown in **Figure 3B**, and the remaining residual structures are shown in **Figure 3A**.

#### 2.2.2 MSF

**Figure 4:**
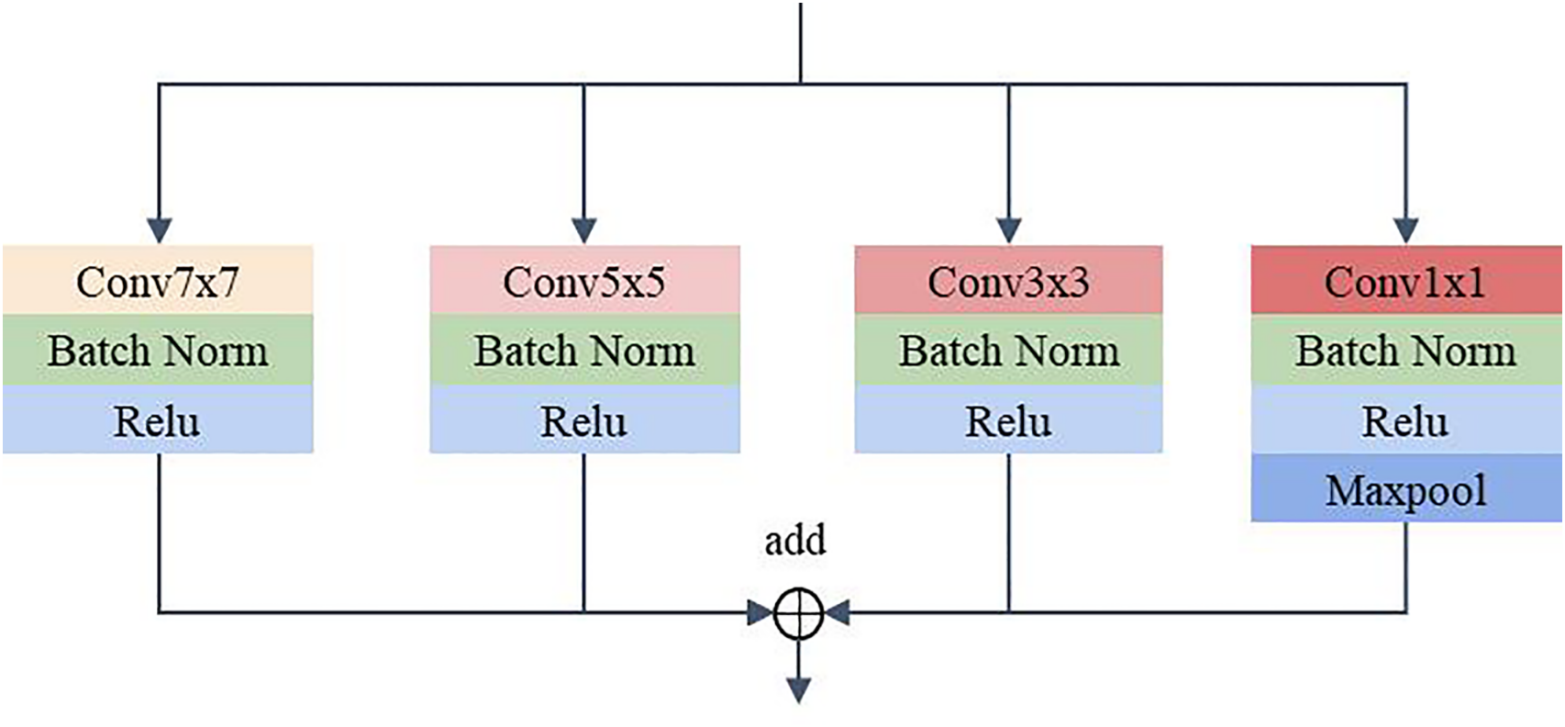
Multiscale feature extraction module.

To address the problem of significant changes in the size and morphology of biological tissues in pathological images, an MSF was developed. It uses multiple scales of convolution kernels to extract multiscale features from histopathological images of bile duct cancer, thus capturing more comprehensive and detailed feature information (25). **Figure 4** shows the details of the MSF. As shown in the figure, convolutional kernel sizes of 3×3, 5×5, 7×7 and 1×1 are used to simultaneously extract feature information from the input image. Each convolution layer contains 64 kernels, and each layer uses a padding strategy to ensure that the input and output feature maps have the same dimension. Except for the 1x1 convolutional kernel, which has stride of 1, the rest of the kernels have a stride of 2. Finally, the feature maps extracted from the convolution of different kernel sizes are output after fusion.

#### 2.2.3 MLF

**Figure 5:**
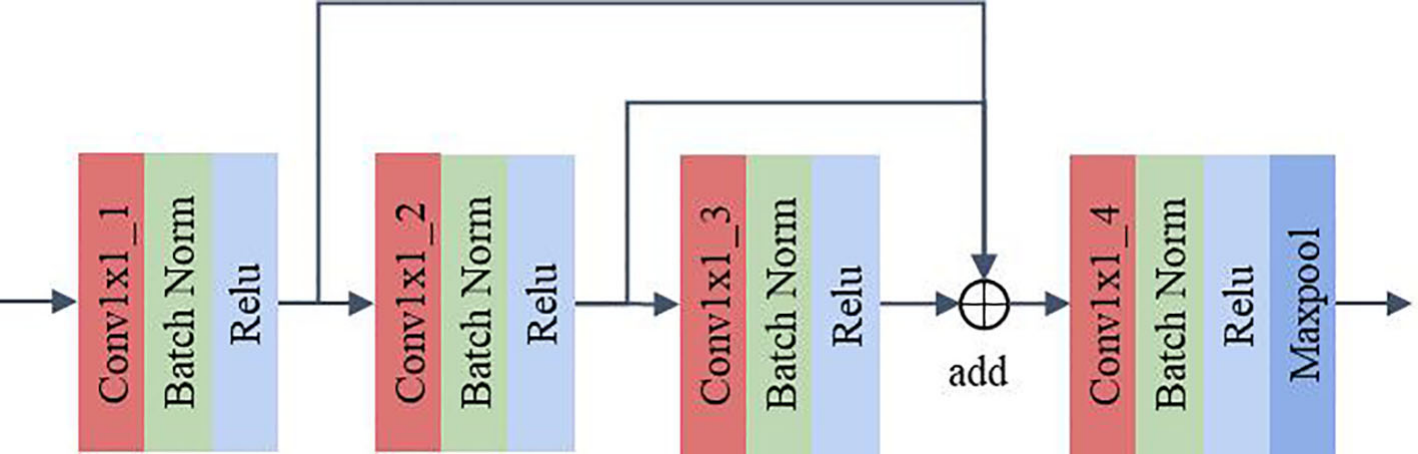
Multilevel feature extraction module.

The MLF uses 1×1 convolution for channel feature extraction and reuses the feature maps extracted from each convolution layer. This design allows the channel features extracted from each convolution layer to be fully utilized without additional computation, which can improve the model’s representation and classification performance. As shown in **Figure 5**, the feature maps pass through Conv1×1_1, Conv1×1_2 and Conv1×1_3 in sequence. The feature maps extracted by the convolution of the above layers are fused and then fed to Conv1×1_4 for feature extraction. The output feature map has twice the number of channels as the input feature map after Conv1x1_4. A maxpool layer with a kernel size of 3×3, stride of 1, and padding of 1 dimensionally reduces the feature map extracted by the convolution layer 4 to obtain the final output. In DCFCNN, 3 multilevel feature extraction modules are used for channel feature extraction. In order to better illustrate the detailed structure of the DCFCNN, the parameters of the overall model are listed in **Table 1**.

**Table 1:** DCFCNN parameters.

Moduel name	Layer (Type)		Filter shape	Input size	Output size
	Resize			2304×1728×3	682×512×3
	CenterCut			682×512×3	448×448×3
MSF	1×1 Conv Maxpool 3×3 Conv 5×5 Conv 7×7 Conv		1×1×64 3×3 3×3×64 5×5×64 7×7×64	448×448×3 448×448×64 448×448×3 448×448×3 448×448×3	448×448×64 224×224×64 224×224×64 224×224×64 224×224×64
MLF_1	1×1 Conv1-4 Maxpool		1×1×64 3×3	224×224×64 224×224×64	224×224×64 112×112×64
MLF_2	1×1 Conv1-4 Maxpool		1×1×128 3×3	112×112×64 112×112×64	112×112×128 56×56×128
MLF_3	1×1 Conv1-4 Maxpool		1×1×256 3×3	56×56×128 28×28×128	56×56×128 28×28×256
RS_1	Residual structure1 Residual structure2		3×3×32;1×1×32 3×3×32	448×448×3 224×224×32	224×224×32 224×224×32
RS_2	Residual structure1 Residual structure2		3×3×64;1×1×64 3×3×64	224×224×32 112×112×64	112×112×64 112×112×64
RS_3	Residual structure1 Residual structure2		3×3×128;1×1×128 3×3×128	112×112×64 56×56×128	56×56×128 56×56×128
RS_4	Residual structure1 Residual structure2		3×3×256;1×1×256 3×3×256	56×56×128 28×28×256	28×28×256 28×28×256
RS_5	Residual structure1 Residual structure2 Residual structure3		3×3×512;1×1×512 3×3×512 3×3×512	28×28×256 14×14×512 14×14×512	14×14×512 14×14×512 14×14×512
	Conv		3×3×1024	14×14×512	14×14,1024
	AdaptiveAvgPool2d		1×1	14×14,1024	1×1024
	Fully connect		1024	1×1024	1×2
			Trainable params: 20,949,378

## 3 Experiment results

This section details the experimental setup, evaluation methods, and results of the proposed method as follows: In Section 3.1 we describe in detail the experimental environment, hyperparameters, and evaluation methods of the proposed method. In Section 3.2, we perform ablation experiments using the proposed method to verify the effectiveness of the modules in DCFCNN.

In Section 3.3, we explore the effect of the fusion level of spatial features and channel features on the final classification results. Section 3.4 shows the effect of resizing and center cropping on the final classification results. Finally, in Section 3.5, the proposed DCFCNN is compared with the classical CNN to validate the advanced classification performance of the proposed method in this paper on the Multidimensional Choledoch Database.

### 3.1 Experimental setup

The method proposed in this paper is based on the Python and Pytorch framework. The experiments were performed on a cloud server with a 14-core Intel(R) Xeon(R) Gold 6330 CPU @ 2.00 GHz, 30 GB RAM, and RTX A5000 24 GB GPU. Five-fold cross-validation was used to prevent bias from different training samples. The distribution of samples in each fold is shown in **Table 2**. The method uses stochastic gradient descent (SGD) to update the weights with a learning rate of 0.001, an impulse of 0.9, and a decay of weights dropoing to 1e-6, a batch size of 16, and an epoch of 200. The weight with the highest overall classification accuracy in the training set was used as the optimal weight for prediction in the test set.

**Table 2:** Five-fold cross-validation sample size distribution.

Fold	K1		K2		K3		K4		K5	
label	0	1	0	1	0	1	0	1	0	1
Train set	113	590	113	590	113	587	114	592	115	593
Test set	29	148	29	148	29	151	28	146	27	145

The evaluation metrics used in this paper include the area under the receiver operating characteristic curve (AUC), accuracy (Acc), sensitivity (SN), specificity (SP), and macro F1-score (F1).

### 3.2 Ablation experiments

In order to verify the effectiveness of the proposed method, this section performs an ablation study on the reuse of features, MSF and MLF, respectively. The relevant experimental results are listed in **Table 3**. In the table, √ means that the module is used, and × means that the module is not used. Note that in experiments 2 and 3, the stride of ResBlock1_1 in the spatial branch is set to 1 to ensure the fusion of spatial features with channel features.

**Table 3:** Ablation experiments.

No	Residual Branch	Feature reuse	Multilevel feature extraction	Multiscale feature extraction	ACC	F1	SN	SP	AUC
1	√	×	×	×	89.98%	80.07%	95.51%	61.16%	0.909
2	√	×	√	×	93.31%	87.35%	96.48%	76.87%	0.944
3	√	√	√	×	93.99%	88.50%	97.01%	78.25%	0.943
4	√	×	√	√	94.43%	89.01%	97.98%	76.03%	0.948
5	√	√	√	√	94.89%	90.10%	97.97%	78.86%	0.952

The experiment is divided into three groups. The first group compares Experiment 1 and Experiment 2 to test the influence of MLF. The second group compared Experiment 2 and Experiment 3, Experiment 4 and Experiment 5 to check the effect of the feature reuse module. The third group compares Experiment 2 and Experiment 4, Experiment 3 and Experiment 5 to verify the impact of MSF.

GROUP 1: The effect of MLF

It is clear from **Table 3** that Experiment 1, which uses a single spatial branch to extract features for classification, has the worst results on all five metrics. Experiment 2 achieved 93.31%, 87.35%, 96.48%, 76.87%, and 0.944 in ACC, F1, SN, SP, and AUC, respectively, after adding the MLF module without feature reuse to extract channel features, which exceeded the classification results of Experiment 1 by 3.33%, 7.28%, 0.97%, 15.71%, and 0.035. It can be seen that better classification performance can be achieved when both deep spatial features and deep channel features are used for classification than when only deep spatial features are used. In other words, MLF contributes to the improvement of classification accuracy.

GROUP 2: The effect of feature reuse

As shown in **Table 3**, the ACC, FC, SN, and SP metrics of Experiment 3 are significantly better than those of Experiment 2, and for the AUC indicators, the difference between the two is very small and can be ignored. Therefore, the classification effect of experiment 3 is better than that of experiment 2. Experiment 4 and Experiment 5 are also similar. The ACC, F, SP, and AUC metrics of Experiment 5 are 94.89%, 90.10%, 78.86%, and 0.952, respectively, exceeding the corresponding metrics 0.46%, 1.09%, 2.83%, and 0.004 of Experiment 4. The SN value, which is 0.01% slightly lower, can also be neglected compared to improving other metrics. The experimental results show that the performance of the model is better after adding the feature reuse module.

GROUP 3: The effect of MSF

From **Table 3**, it can be seen that, with the exception of the SP index of Experiment 4, which is 0.84% lower than that of Experiment 2, the other four metrics are all better than those of Experiment 2. The comparison between Experiment 3 and Experiment 5 is also very similar. All of the metrics from Experiment 5 are better than Experiment 3. ACC, F1, SN, SP, and AUC increased by 0.9%, 1.6%, 0.87%, 0.61%, and 0.009, respectively. Therefore, the above experimental results demonstrate the effectiveness of MSF in classification.

**Figure 6:**
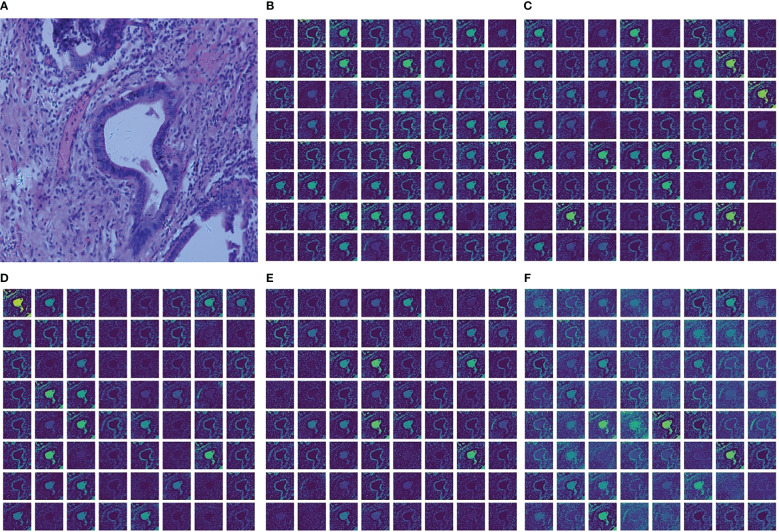
Cholangiocarcinoma images and visualization of feature maps in MSF. **(A)** Cholangiocarcinoma images; **(B)** Feature maps extracted from Conv1x1; **(C)** Feature maps extracted from Conv3x3; **(D)** Feature maps extracted from Conv5x5; **(E)** Feature maps extracted from Conv7x7; **(F)** The final output feature maps.

To better illustrate the performance improvement achieved by MSF, this paper shows the feature maps captured from each convolutional layer in MSF and the final output feature map of MSF in **Figure 6**. The brightness of the image represents the information in which the convolution kernels are interested. The higher the brightness, the more information the convolution kernels focus on and learn from the area. The lower the luminance, the less attention the kernel pays to the information contained in the region, and the less information it learns. **Figure 6A** shows the histopathological image of a cholangiocarcinoma. **Figures 6B–F** shows the feature maps extracted from the convolution kernels of 1×1, 3×3, 5×5, 7×7 and the final output feature maps after fusing feature maps extracted from different convolution layers. Comparing **Figure 6F** with **Figures 6B–E**, it can be found that compared with the feature maps extracted from a single-size convolutional layer, there are fewer low-brightness feature maps in the fused feature map, and there are significantly more “bright regions” in each feature map of **Figure 6F**. This means that MSF helps the model capture richer feature information from the original Cholangiocarcinoma histopathological images. It should be noted that although the increase of “bright areas” can bring more abundant feature information, not all feature information is beneficial to the model. This puts forward higher requirements for the design of the subsequent channel feature extraction module. MLF with the feature reuse module reuses the feature information extracted in each convolutional layer to obtain a better feature representation, thereby suppressing the noise and redundancy caused by MSF.

The above experimental results show that regardless of MSF, MLF, or feature reuse, they all contribute to the improvement of the classification accuracy of the cholangiocarcinoma histopathology image dataset.

### 3.3 Effect of fusion layers on classification results

**Figure 7:**
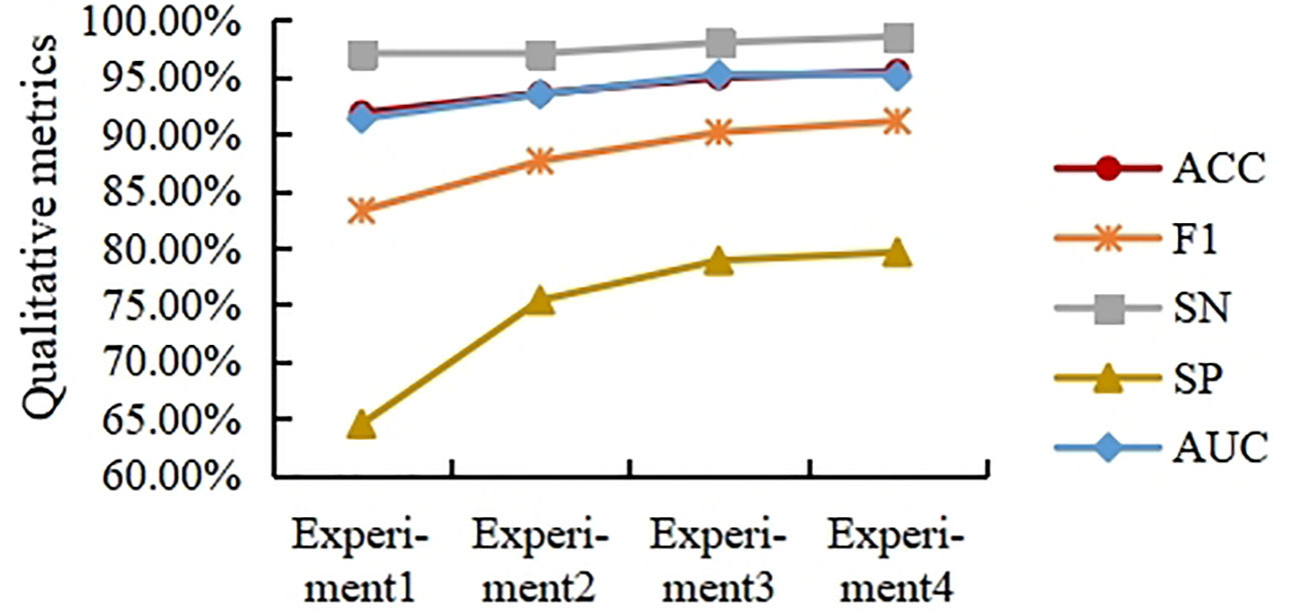
Experimental results of different fusion layers.

This section studies the influence of spatial and channel features on the final classification results when merged at different layers. For this purpose, four experiments were designed. In experiments 1 to 4, the spatial branches remain the same, and the number of MLFs in the channel branches is adjusted. Specifically, Experiment 1 contains one MLF, Experiment 2 contains two MLFs, Experiment 3 contains three MLFs, and Experiment 4 contains four MLFs. Note that in Experiment 4, the number of channels of the last MLF is 512. The experimental results are shown in **Figure 7**.

As shown in **Figure 7**, the ACC, F1, SN, SP, and AUC curves generally showed an upward trend with increasing fusion level, but the trend slowed down with increasing fusion level. Overall, in the task presented in this paper, the performance of fusing deep spatial features with deep channel features is higher than that of fusing shallow spatial features with shallow channel features. In this paper, the fusion strategy in Experiment 3 is selected from the viewpoint of saving computation costs.

### 3.4 Effect of different resize and center crop sizes on classification results

In order to solve the problem of the extremely high spatial dimensionality of the histopathological images of cholangiocarcinomas, resize transformation and center cropping are used in the method proposed here. However, while the above operation results in a reduction in the dimensionality of the spatial input, it also results in a loss of feature information. To measure the effect of different resizing and center cropping sizes on the classification performance of the model, four experiments are performed in this section. In Experiment 1, the short edges were first scaled to 256 (the long edges were scaled the same), and the input image was then spatially sized to 224×224 using center cropping. For the sake of simplicity, this process is written as 256-224 in this paper. Experiment 2 is 384-336, Experiment 3 is 512-448 and Experiment 4 is 640-560. To avoid misleading expressions in section 3.3, we use 256-224, 384-336, 512-448 and 640-560 to represent experiment 1, 2, 3, and 4, respectively, in this section.

**Figure 8:**
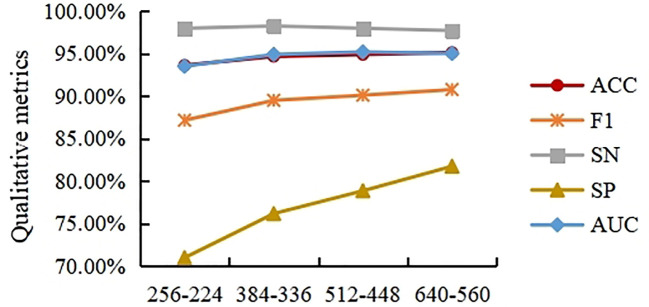
Experimental results on different of cholangiocarcinoma histopathological image sizes.

As shown in **Figure 8**, the ACC, F1, and AUC curves roughly show an increasing trend, but the trend tends to weaken as the size of the input image increases. This indicates that modestly increasing the input image size can help improve the model’s classification accuracy for ACC, F1, and SP. However, excessively increasing the input image size does not bring significant improvements in ACC, F1, or SP. In general, excessive resizing and center cropping will lose most of the feature information in the original image, reducing the model’s classification performance. Taking into account the computational cost of the model, this paper selects the resizing and center cropping strategy of 384-336 mentioned above, where the short edges were first scaled to 384 (the long edges were scaled the same), and the input image was then spatialized to 336×336 using center cropping.

### 3.5 Comparison with other methods

In this section, the DCFCNN is compared to four classic convolutional neural networks, including AlexNet, Vgg19_bn, ResNet152, and DesNet161, in the Multidimensional Choledoch Database. AlexNet is a milestone algorithm for deep learning. Though overtaken by more efficient architectures, it is an important step from shallow to deep networks, and its proposed dropout, ReLU, and preprocessing are still the key steps for many improved algorithms. VGG is a successor to AlexNet. It is a deep convolution model based on small convolutional kernels. This type of model includes Vgg 16, Vgg 19, Vgg 16_bn, and Vgg 19_bn. Vgg19_bn is a VGG 19-layer model with batch normalization that typically gives better results for most image classification tasks. ResNet is a short name for a residual network. It has fewer filters and less complexity than VGG nets. Each ResNet block is either two layers deep (used in small networks such as ResNet 18, 34) or three layers deep (ResNet 50, 101, 152). The deeper ResNet achieves better training results as compared to the shallow network and significantly outperforms it when the network is deeper. Therefore, we chose ResNet152 in our experiments. DesNet is a densely connected convolutional network based on short paths and feature reuse. Considering the size and accuracy of the model, the Densenet 161 model was chosen for our experiments. All of the above networks are pre-processed in the same way. That is, they go through the same four steps: resizing, center cropping, normalization, and standardization. The learning rate, weight decay, momentum, and batch size are consistent with the method proposed in this paper.

**Table 4** shows the ACC, F1, SN, SP, and AUC values of different models, as well as the training and test time. As can be seen from the table, AlexNet is a shallow structure compared to other models, so its five metrics are all the worst. Vgg19_BN improves classification performance by increasing network depth. Classification accuracy is higher for the five metrics than AlexNet. The ResNet152 model uses residual learning to alleviate network degradation and overfitting problems, so Acc, F1, SP, and AUC are further improved, but the SN metric is slightly lower than Vgg19_bn. DesNet161 deepens the network structure while enhancing the model representation, so the classification performance is improved. However, this method ignores channel features, so its classification performance is lower than that of the DCFCNN proposed in this paper. The ACC, F1, SP, and AUC values of the DCFCNN model are better than those of the DesNet161 model, which are 0.45%, 1%, 2.71%, and 0.006 higher, respectively. The SN indicators are equivalent to 97.97%.

**Table 4:** Experimental results of different methods on Multidimensional Choledoch Database.

Network	Evaluation						
	Acc	F1	SN	SP	AUC	Train time(s)	Test times(s)
AlexNet	85.54%	73.00%	91.19%	56.08%	0.821	2668.98	6.79
Vgg19_bn	91.14%	81.43%	97.43%	58.50%	0.901	4187.19	7.82
ResNet152	92.04%	84.42%	96.47%	69.10%	0.904	4453.33	9.11
DesNet161	94.44%	89.10%	97.97%	76.15%	0.946	4988.35	12.54
DCFCNN	94.89%	90.10%	97.97%	78.86%	0.952	2975.05	8.27

The training time and test time of each model are also shown in **Table 4**. Although the network depth of DesNet161 is similar to ResNet152, its training time and testing time are the longest among all models. Compared with the DCFCNN, Vgg19_bn takes less test time but much longer training time. Although AlexNet’s test time and training time are lower than DCFCNN’s, the classification performance of DCFCNN is much better than AlexNet’s. In general, the proposed DCFCNN model has the best comprehensive performance.

## 4 Conclusion

In this paper, we propose a histopathological image classification method for cholangiocarcinoma based on a convolutional neural network with spatial-channel feature fusion. In particular, both spatial and channel features are used to classify histopathological images of cholangiocarcinoma, and a multiscale feature extraction module is used to obtain richer features from the original image. In addition, multi-level feature extraction modules are designed to take full advantage of the channel features extracted from different convolutional layers to enhance the model representation. The results of a multidimensional cholangiocarcinoma dataset show that the proposed method outperforms the widely used classical CNN model.

Cholangiocarcinoma has three distinct stages of differentiation: low, intermediate, and high. The method in this paper only focuses on classifying cancerous and non-cancerous images. In future research, we can make a more detailed classification based on the differentiation of different stages. Furthermore, a multi-branch, multi-level fusion strategy will be introduced to further investigate the influence of spatial features and channel features on the final classification performance under different fusion states. In addition, it is assumed that attentional mechanisms and a long short-term memory should be introduced to explore the local channel features.

## Data availability statement

The original contributions presented in the study are included in the article/supplementary material. Further inquiries can be directed to the corresponding authors.

## Author contributions

HZ and ZY contributed to conception and design of the study. HZ conducted most of the experiments, performed the statistical analysis and wrote the first draft of the manuscript. JL organized the data and gave suggestions for improvement. JH conducted parts of the experiments. All authors contributed to the article and approved the submitted version.
